# Disulfiram Enhances the Antineoplastic Activity and Sensitivity of Murine Hepatocellular Carcinoma to 5-FU via Redox Management

**DOI:** 10.3390/ph16020169

**Published:** 2023-01-23

**Authors:** Iftekhar Hassan, Hossam Ebaid, Ibrahim M. Alhazza, Jameel Al-Tamimi, Ahmed M. Rady

**Affiliations:** Department of Zoology, College of Science, King Saud University, Riyadh 11451, Saudi Arabia

**Keywords:** liver, cancer, copper, 5-FU, disulfiram, apoptosis

## Abstract

The efficacy of anticancer drug 5-FU is suppressed due to various factors, including severe side effects and decreased insensitivity during prolonged chemotherapy. Elevated endogenous copper (Cu) levels are one of the prominent hallmark features of cancer cells. In the present investigation, this feature was targeted in diethyl nitrosamine-phenobarbital-induced hepatocellular carcinoma (HCC) in a rat model system by an established anticancer drug, 5-FU, co-administered with copper and its chelating agent, disulfiram. After treatment with the test chemicals in HCC-induced rats, blood and liver samples were subjected to biochemical, molecular, and histopathological analyses. The analysis revealed that reactive oxygen species-mediated oxidative stress is the crucial etiological reason for the pathogenesis of HCC in rats, as evidenced by the significantly compromised activity of major antioxidant enzymes and elevated levels of oxidative damaged products with major histological alterations compared to the control. However, the combination of 5-FU with DSF demonstrated a significant improvement in most of the parameters, followed by 5-FU-Cu in the combination-treated groups. The combination treatment improved the histological details and triggered apoptosis in the cancer cells to a remarkable extent, as the levels of cleaved PARP and caspase-3 were significantly higher than those in the HCC rats treated with the drug alone. The present study envisages that manipulating the Cu-level greatly enhances the antineoplastic activity of 5-FU and sensitizes cancer cells to the increased efficacy of the drug.

## 1. Introduction

Cancer is a heterogeneous and multistage disease resulting from uncontrolled cellular proliferation (initiation), consequently leading to the invasiveness of neighboring cells (progression) and distant healthy cells (metastasis). These tumorous cells can ditch cell death signals and exhaust the body’s immune system [1,2]. Despite significant developments in cancer treatment and oncology over the last five decades, the disease is still considered the second most fatal after cardiovascular diseases. Currently, a variety of cancer treatment modalities are available, including surgery, chemotherapy, radiotherapy, immunotherapy, hormonal therapy, catalytic therapy, and gene therapy, but chemotherapy based on antineoplastic drugs/agents alone or in combination is still the most favored and effective treatment strategy among all [3]. Despite developing all these treatment strategies, they are limited mainly by either their short-lived efficacy or the treatment regimen being modified or discontinued because of associated mild to severe side effects [4]. Hence, combining two or more treatment modalities is considered the best strategy to contain the disease.

Copper (Cu) is an essential metal-nutrient requisite for all life forms. The metal is incorporated into several metalloenzymes and metalloproteins involved in diverse biological activities. It executes a vital role in various metabolic and physiological processes, such as metabolism, nerve induction, neurotransmission, erythropoiesis, connective tissue synthesis, assistance to all iron-mediating biological actions in addition to immunity boost-up, including angiogenesis, wound healing and pathophysiology involving tumor growth and inflammatory diseases [5,6,7]. Cu-dependent enzymes, such as cytochrome c oxidase, NADH dehydrogenase-2, Cu/Zn superoxide dismutase, tyrosinase, ferroxidases, monoamine oxidase, and dopamine β-monooxygenase, reduce molecular oxygen. The unique redox property of Cu that enables it to deliver various biological activities also poses a potentially toxic effect because its ambivalent effects result in the generation of invasive free radicals such as superoxide and hydroxyl radicals [8,9]. If not properly regulated in the living system, copper can exert extreme cytotoxic effects and genetic mutations that can compromise Cu-homeostasis and be translated into severe clinical phenotypes. Therefore, understanding how cells maintain optimal copper levels is highly relevant to human health [10].

As one of the most prominent hallmarks in cancerous cells, the elevated endogenous Cu level has drawn the interest of many contemporary researchers [11]. In addition, Cu induces neovascularization, and its concentration is profoundly increased in angiogenic tissue [12]. Cu chelators, used to treat Wilson disease (a disease of Cu toxicity), have been cited for inhibiting tumor growth and angiogenic responses in many studies [7]. Moreover, Cu also plays a vital role in inflammatory responses involved in innate and adaptive immunity by activating NF-κB [13]. Alternatively, Cu deficiency alters the intravascular adhesion of leukocytes to activated ECs and the expression of adhesion molecules, such as ICAM-1/VCAM-112,5,13 [14]. Despite the critical role of Cu in angiogenesis and inflammation, the exact molecular mechanisms underlying these functions are entirely unknown.

Oxidative stress is a disparity between the generation of highly reactive free radicals and metabolites (oxidants) and their nullification by the natural machinery (antioxidant system) in living organisms. This imbalance causes damage to biologically significant biomolecules (proteins, lipids, and nucleic acids) and a cellular structure that can subsequently compromise cellular function and integrity. Moreover, stress can lead to chronic inflammation induced by free radicals and concurrent biological, chemical, and physical factors that enhance the chances of serious diseases, including several types of human cancers [15]. Hence, there is a strong association between oxidative stress, inflammation, and carcinogenesis, as shown by various epidemiological and experimental studies [16,17]. Hence, many antioxidants and anti-inflammatory therapies show significant efficacy in cancer prevention and management [18]. Virchow was the first to plead that certain types of inflammatory cells occur within tumors and that most tumors originate at sites of chronic inflammation [19]. Hence, such inflammation is often referred to as the “secret killer” for many health-related issues and the etiology of many diseases, such as cancer [20]. If this is not regulated, a potentially negative impact falls on the whole organism, resulting in various diseases [21]. Therefore, a vivid relationship between oxidative stress, inflammation, and cancer has recently been more widely accepted among contemporary researchers and oncologists [20].

Cancer therapeutic resistance is supposed to be directly related to the disease’s late stages and metastasis phase; however, there are no effective interventions to prevent cancer progression at such stages, even with the latest combinations of treatment modalities. Therefore, searching for novel compounds of therapeutic potential and suggestions for anticancer treatment strategies that might pave the way for a fruitful breakthrough in the future become a necessity [21]. We have previously found that endogenous copper is critical in determining the efficacy and outcome of chemotherapeutic agents [2]. This study examines whether the level of endogenous Cu plays a major role in attributing therapeutic interventions against tumors by investigating oxidative stress and critical apoptotic markers. In this work, we tried to explore how the manipulation of Cu levels can influence the anticancer efficacy of 5-FU in DEN-induced hepatocellular carcinoma in a rat model system.

## 2. Results

### 2.1. Effect on Cancer Markers

#### 2.1.1. AFP

**Figure 1:**
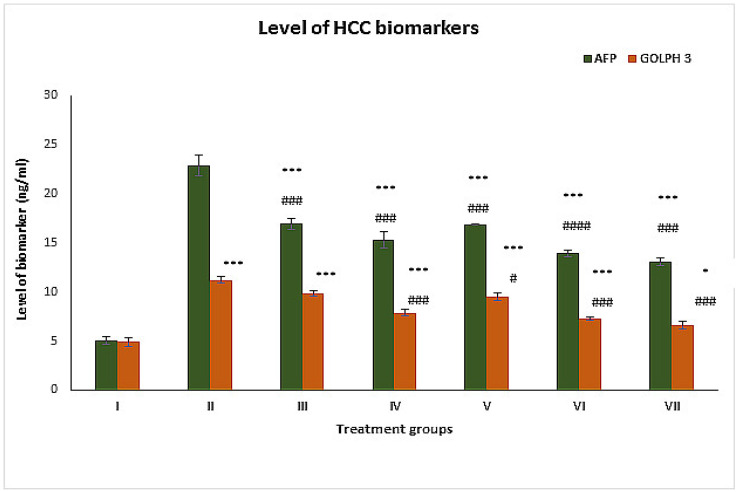
Levels of hepatocellular carcinoma markers alpha-fetoprotein (AFP) and Golgi phosphoprotein 3 (GOLPH 3) in the serum samples of the indicated groups expressed as nanograms per milliliter of sample. The asterisk mark indicates values significantly different from the control (group I), while hashtag indicates values significantly different from the control positive (group II). All experiments were conducted in triplicate, and the data are presented as the mean ± SEM. *p*-values were calculated by Student’s *t*-test, * or # *p* < 0.05; *** or ### *p* < 0.001.

This is believed to be one of the most reliable cancer markers. It was elevated by 352.37% in group II compared to the control group. Groups I, IV and V demonstrated a decrease in its level by 25.90%, 32.91%, and 26.21%, respectively, compared to group II. Among the combination groups, groups VI and VII showed a decline in their levels of 39% and 42.88%, respectively, compared to group II (Figure 1).

#### 2.1.2. GOLPH 3

This is also a very accurate biomarker to assess hepatocellular carcinoma. In the present investigation, group II exhibited a rise in its level of 128.97% compared to group I. Groups III, IV and V demonstrated a decline in their level by 12.29%, 29.85% and 14.88%, respectively. In contrast, groups VI and VII showed a dip in their level by 35.02% and 40.90%, respectively, compared to group II (Figure 1).

### 2.2. Effect on Oxidative Stress Parameters

#### 2.2.1. SOD

**Figure 2:**
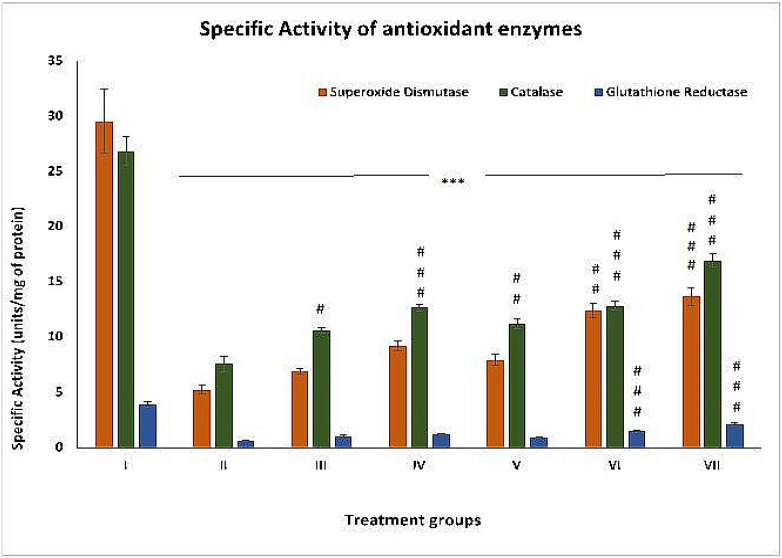
Effect of the treatment on the specific activity of antioxidant enzymes—copper-zinc superoxide dismutase (SOD), catalase (CAT), and glutathione reductase (GR) in liver samples of the indicated treatment groups expressed in units per milligram of protein in the sample. The asterisk mark indicates values significantly different from the control (group I), while hashtag indicates values significantly different from the control positive (group II). All experiments were conducted in triplicate, and the data are presented as the mean ± SEM. *p*-values were calculated by Student’s *t*-test, # *p* < 0.05; *** *p* < 0.001.

This is assumed to be one of the chief antioxidant enzymes in our body. The specific activity of this enzyme was compromised by 82.19% in group II compared to group I. Groups III, IV and V showed an increase in the activity by 31.55%, 76.23%, and 50.95%, respectively. In comparison, groups VI and VII demonstrated enhancements in activity by 135.74% and 160.26%, respectively, compared to group II (Figure 2).

#### 2.2.2. CAT

The specific activity of this antioxidant enzyme was decreased by 71.73% in group II compared to group I. Groups III, IV and V exhibited increases in its activity by 40.05%, 67.06% and 48.08%, respectively. In contrast, groups VI and VII showed elevations in activity by 69.56% and 123.45%, respectively, compared to group II (Figure 2).

#### 2.2.3. GR

The specific activity of this enzyme decreased by 84.71% in group II compared to group I. Groups III, IV and V showed an increase in its activity by 68.12%, 94.37%, and 46.87%, respectively. In comparison, groups VI and VII exhibited enhancements in activity by 137.5% and 235.93%, respectively, with respect to group II (Figure 2).

#### 2.2.4. MDA Levels

**Figure 3:**
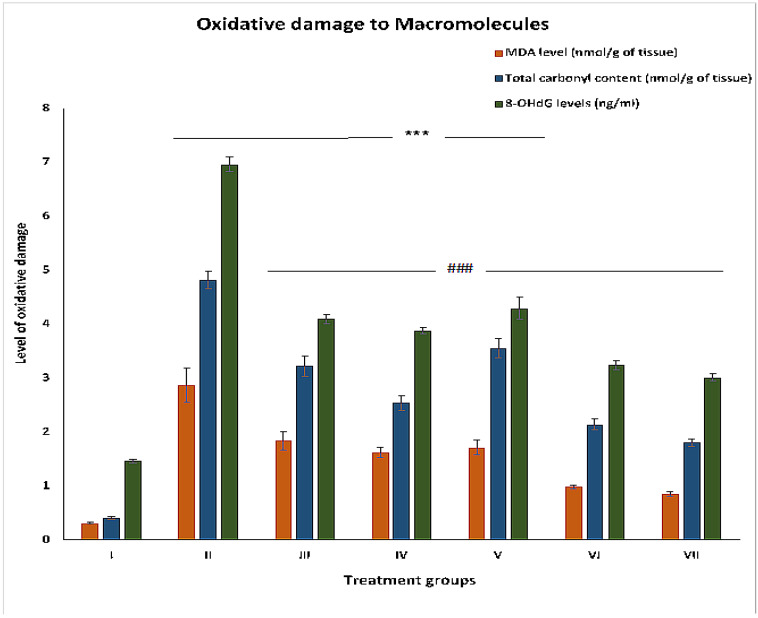
Level of the products of oxidative damage to macromolecules (malondialdehyde; MDA, carbonyl content, 8-hydroxy-2’-deoxyguanosine;8-OHdG) expressed in the indicated units in parentheses in the liver samples. The asterisk mark indicates values significantly different from the control (group I), while hashtag indicates values significantly different from the control positive (group II). All experiments were conducted in triplicate, and the data are presented as the mean ± SEM. *p*-values were calculated by Student’s *t*-test, *** or ### *p* < 0.001.

MDA is considered the most stable final product after lipid peroxidation in vivo. Its level was staggeringly high in group II, i.e., by 825.80% compared to group I. Other groups III, IV and V showed a decrease in its level by 35.88%, 43.55% and 40.42% compared to group II. However, the combination groups VI and VII demonstrated reduction in their levels by 65.85% and 70.38%, respectively, compared to group II (Figure 3).

#### 2.2.5. Total Carbonyl Content

Group II showed a sharp increase in its level by 1075.60% compared to group I. Groups III, IV and V displayed declines of 33.19%, 47.30%, and 26.34%, while groups VI and VII showed decreases of 55.60% and 62.65%, respectively, in comparison to group II (Figure 3).

#### 2.2.6. 8-OHdG

This is one of the reliable markers to assess oxidative DNA damage and the extent of carcinogenesis. Its level was increased by 376.71% in group II with respect to group I. Groups III, IV, and V showed a decrease in its level by 41.23%, 44.39% and 38.36%, respectively, compared to group II. The combination groups VI and VII demonstrated dips in their levels by 53.44% and 56.72% compared to group II (Figure 3).

### 2.3. Effect on Apoptotic Markers

#### 2.3.1. p-53

**Figure 4:**
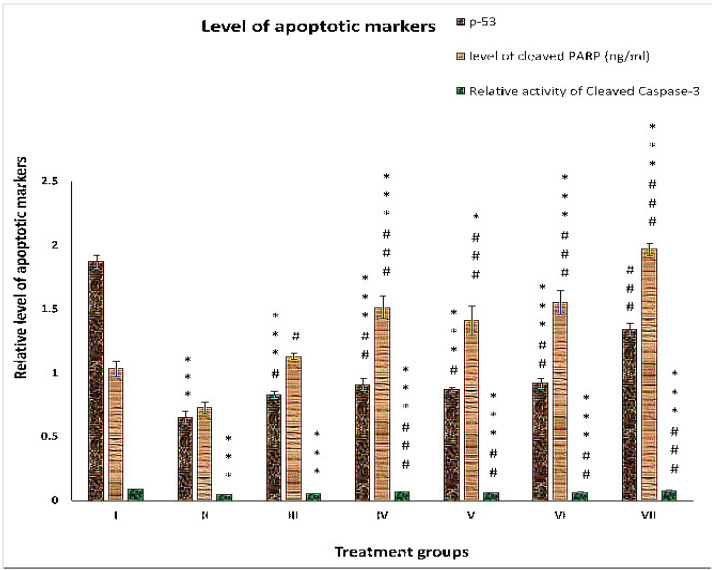
Relative levels of apoptotic markers (p-53, cleaved caspase-3, and cleaved PARP) in the liver samples of the treated groups. The asterisk mark indicates values significantly different from the control (group I), while hashtag indicates values significantly different from the control positive (group II). All experiments were conducted in triplicate, and the data are presented as the mean ± SEM. *p*-values were calculated by Student’s *t*-test, * or # *p* < 0.05.

This is considered the guardian gene in the nucleus of a cell that stabilizes nuclear DNA and its integrity. In the present study, group II showed a 65.24% decline in its level compared to group I. Groups III, IV and V demonstrated 27.69%, 40%, and 33.84% enhancements of its level, while groups VI and VII exhibited 41.53% and 106.15% increases in its level, respectively, compared to group II (Figure 4).

#### 2.3.2. Cleaved PARP

This is one of the most prominent markers to confirm the occurrence of apoptosis. Its level was decreased by 29.66% in group II compared to group I. Groups III, IV and V showed an increase in its level by 55.35%, 107.82%, and 94.23%, while group VI and VII displayed a rise in their level by 113.05% and 170.46% compared to group II (Figure 4).

#### 2.3.3. Cleaved Caspase-3

The level of cleaved caspase-3 was compromised by 46.73% in group II compared to group I. Groups III, IV, V, VI and VII showed increases in cleaved caspase-3 levels of 16.32%, 42.85%, 26.53%, 32.65% and 65.30%, respectively, compared to group II (Figure 4).

### 2.4. Effect on Nuclear DNA of the Liver Cells

**Figure 5:**
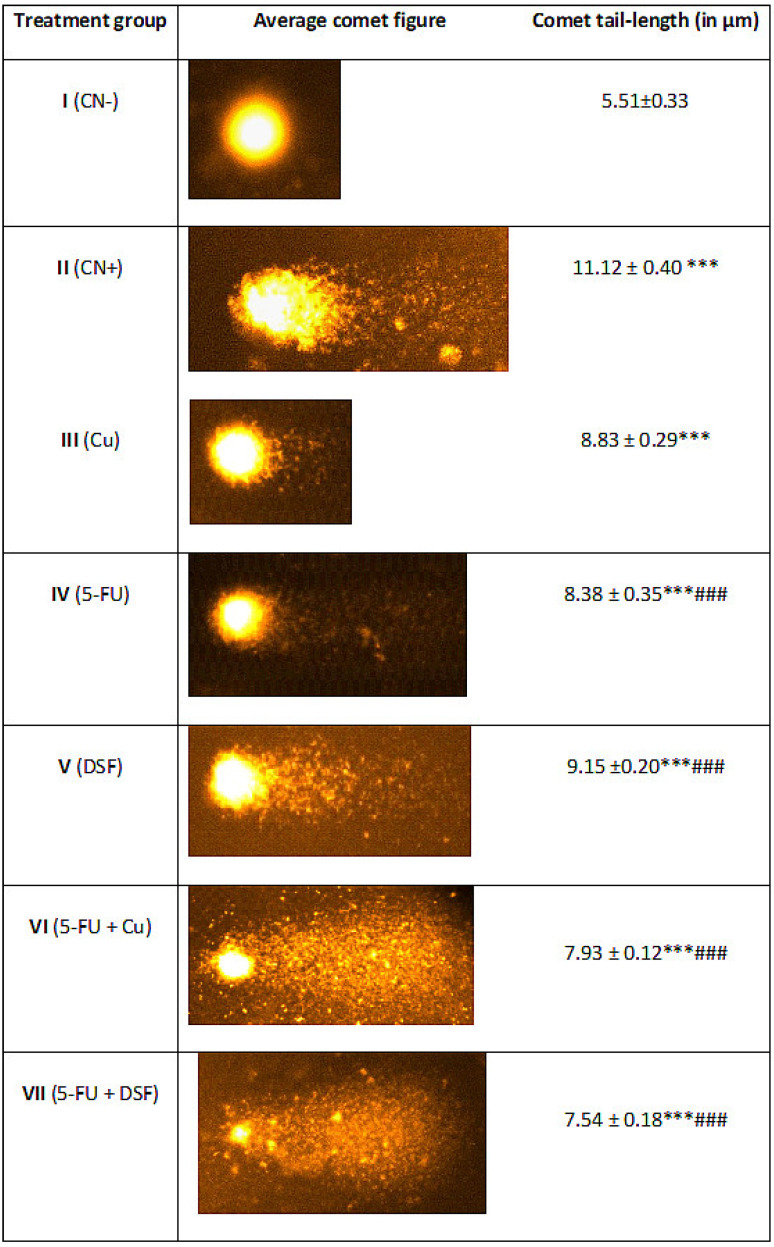
Average picture of the comet assay of liver cells of the treated groups. The comet tail lengths are in µm. The asterisk mark indicates values significantly different from the control (group I), while hashtag indicates values significantly different from the control positive (group II). All experiments were conducted in triplicate, and the data are presented as the mean ± SEM. *p*-values were calculated by Student’s *t*-test, *** or ### *p* < 0.001.

In the present work, group II demonstrated an increase in tail length by 101.08% compared to the control. However, group III, IV, V, VI and VII showed a decrease in tail length by 20.59%, 24.64%, 17.71%, 28.68% and 32.19%, respectively, when compared to group II (Figure 5).

### 2.5. Assessment of Necrosis by LDH Activity

**Figure 6:**
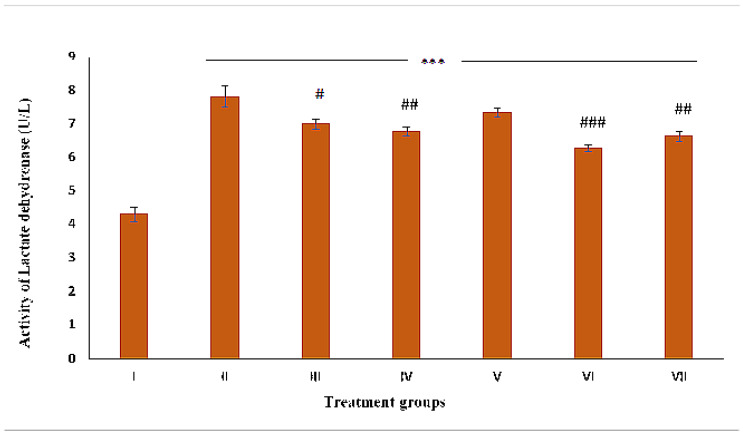
Bar diagram of the level of lactate dehydrogenase (LDH) in the liver samples. The activity of the enzyme is expressed in units/liter. The asterisk mark indicates values significantly different from the control (group I), while hashtag indicates values significantly different from the control positive (group II). All experiments were conducted in triplicate, and the data are presented as the mean ± SEM. *p*-values were calculated by Student’s *t*-test, # *p* < 0.05; ## *p* < 0.01; *** or ### *p* < 0.001.

The activity of group II was enhanced by 82.05% compared to the control. Groups III, IV, V, VI and VII demonstrated a decrease in its activity by 10.49%, 13.44%, 6.14%, 15.36% and 19.71%, respectively, compared to group II (Figure 6).

### 2.6. Histopathological Evaluation

**Figure 7:**
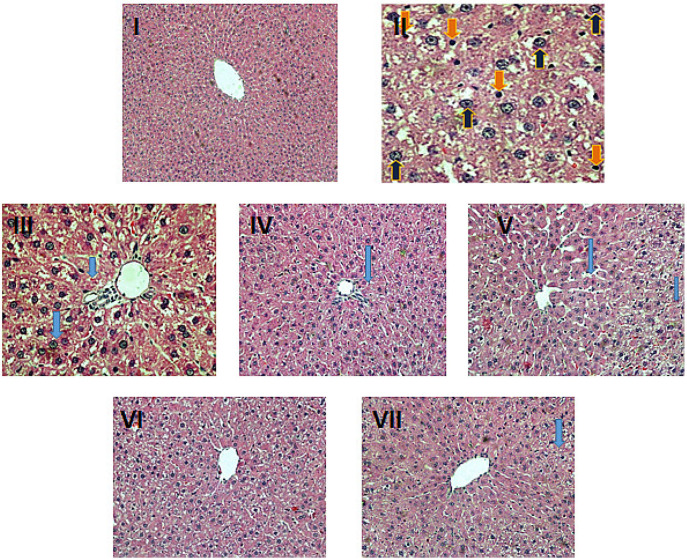
Histomicrographs of liver samples of the indicated groups in parentheses. All sections were stained with hematoxylin and eosin and snapped at 400× (Leica, Munich, Germany).

The histological evaluation of the liver samples from the treatment groups reflected marked differences among the various groups, confirming the biochemical and oxidative stress analysis. The histology of the control group, group I, showed typical histological features of a normal liver with well-maintained contours of healthy hepatocytes with normal nuclei radiating toward the central vein. The less developed tumor nodules/patches on the color faded surface of the liver in the DEN-PB-treated positive control (group II) indicated hepatocellular carcinoma in the treated rats (pictures not shown). The histopathology of the control-positive group II showed severe histological alterations in the hepatocytes, mostly with distorted contours, extensive vacuole formation, disturbed sinusoids and inflammatory infiltration indicative of partial loss of tissue in the form of fibrosis and necrosis in addition to apoptosis in some of the cells. The other groups, III and V, demonstrated mild to moderate damage, including light inflammatory cell infiltration and unclear cellular boundaries with narrow sinusoids; however, these features were less prominent in group IV. The combination groups VI and VII showed improved histological details, including less vacuole formation and ceasing inflammatory features with less fibrosis and normal sinusoids with normal nuclei; however, group VII showed better histological restoration than group VI (Figure 7).

## 3. Discussion

Hepatocellular carcinoma (HCC) is the fifth most common form of cancer and causes the death of almost 600,000 patients around the world annually [22]. This form of cancer arises from complicated and multistage carcinogenesis after chronic exposure to several mitogenic and mutagenic agents, causing random genetic alterations, consequently leading to this phenotype as a disease [2]. This cancer has a high mortality rate, as its diagnosis is difficult at the early stage. Despite being a widely occurring form of cancer, no concrete breakthrough has been achieved yet for its effective treatment, even after many decades of research. However, the endogenous Cu level was significantly elevated in HCC-induced rats, similar to many other types of cancer [23]. The present investigation aims to target endogenous Cu levels to enhance the antineoplastic activity of 5-FU in HCC-induced rats, which might be an effective strategic cancer management strategy for prolonging the life span and quality of life.

**Figure 8:**
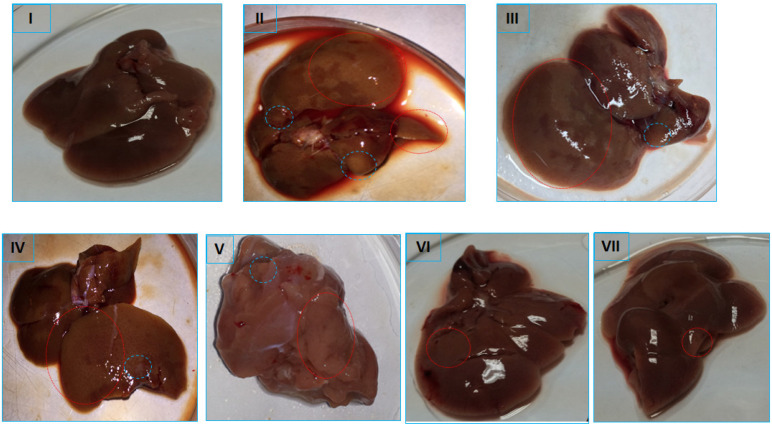
Average picture of liver from the treated groups highlighted damaged portion of the organ.

In the present study, we found that DEN-PB was able to induce the second stage of HCC, as evidenced by biochemical analysis showing staggeringly enhanced tumor markers (AFP and GOLPH3) and compromised antioxidant parameters (SOD, CAT, and GR) concomitant with highly elevated products of oxidative damage to key macromolecules (MDA, carbonyl content and 8-OHdG). Furthermore, the histopathological evaluation revealed an extensive alteration in the structure of hepatocytes and distorted sinusoids, indicating acute inflammation, fibrosis, and even necrotic signs. The rats treated with copper, 5-FU and DSF also showed mild to moderate perturbation in their biochemical analysis, antioxidative parameters, oxidative stress-induced damage, and histological assessment. However, the 5-FU-treated group showed improvement in most parameters compared to the other two groups (Cu and DSF). However, the combination of the drug with Cu and DSF showed stronger antineoplastic activity than 5-FU alone in HCC rats. These combinations decreased the burden of cancer in the treated rats and improved antioxidant parameters with a significantly diminished level of oxidative damage to lipids, proteins, and DNA. It is also noteworthy that the combination of 5-FU + DSF showed better antineoplastic activity than 5-FU + Cu in the present investigation (Figure 8).

It is well established that reactive oxygen and nitrogen species (ROS and RNS), which cause relative oxidative and nitrosative stress in vivo, are the major cause of various physiological alterations, immunological pathogenesis, and diseases, including cancer [19]. In the present study, ROS and oxidative stress also play a pivotal role in chemically induced HCC, which is quite evident from the compromised activity of antioxidant enzymes concomitant with significantly elevated levels of MDA, carbonyl content and 8-OHdG in the cancer-induced group. Furthermore, endogenous Cu is generally elevated in cells under conditions that also facilitate ROS-mediated cellular and macromolecular damage in their vicinity. These reactive species are invasive enough to cross through the nucleus and interact with nuclear DNA, which might affect major genes, including p-53 and p-21 [23]. This might be one of the attributes of genetic instability in cancer cells, as indicated by elevated 8-OHdG levels in the HCC group. In addition, this genetic alteration can affect the normal cell cycle of such cells, resulting in necrosis or apoptosis depending on the severity of the damage incurred. In contrast, the Cu-treated rats showed slight improvement in many of the parameters that might be chiefly attributed to the enhanced activity of the antioxidant enzyme Cu-Zn-SOD in addition to the metal supporting the chromatin of nuclear DNA. It has been reported that administered Cu can bind with metallothionein (MT), accumulating in cancer cells’ lysosomes and increasing their susceptibility to cell death. In addition, the metal can also pass through the mitochondrial membrane of the cells, altering the organelle functions and gene expression that might result in apoptosis and necrosis [24,25]. However, DSF treatment showed a better effect in HCC rats than Cu treatment. The chelating agent seems to lower oxidative stress and the related damage incurred by excessive endogenous Cu in cancer cells. In addition, much of the literature suggests that the DSF-Cu complex exerts potent proteasome inhibitory and apoptosis-inducing effects in Cu-rich tumor cells with precise target specificity [26]. Furthermore, Denoyer et al., [27] reported that DSF, a member of the dithiocarbamate family, triggers a series of events, such as affecting the redox-related cellular machinery, altering cellular glutathione and other redox thiol proteins and changing mitochondrial permeability. All these events eventually lead to apoptosis induction in the affected cells [28].

The present investigation is based on hypothesis Hassan et al. [2]: that if the elevated endogenous Cu of the cancer cells is restraint managed, they can be converted into bullets to kill the same cells. Interestingly, co-administered Cu and DSF with the anticancer drug 5-FU showed enhanced antineoplastic activity compared to the drug alone in chemically induced HCC rats. As excessive Cu levels are one of the major causes of drug resistance, the 5-FU-DSF combination showed better efficacy than the 5-FU-Cu combination. Many earlier studies have demonstrated that DSF at the appropriate dose can induce apoptosis by a staggering 400–600% in many cell lines and animal model-based studies [29,30]. Additionally, Chen et al. [29] demonstrated that DSF not only triggers apoptosis but also enhances the apoptosis susceptibility of tumor cells in vitro and in vivo. The chelating agent has been reported to trigger apoptosis in cancer cells by various modes of action, including depleting the GSH level, decreasing the mitochondrial membrane potential and transiently increasing the cellular superoxide level [2]. Moreover, DSF also orchestrates the immune system and ceases the inflammatory and aggressive immune response in the affected cells [26]. In addition, cleaved PARP and cleaved caspase-3 were significantly higher in 5-FU-DSF-treated rats than in 5-FU-Cu-treated rats. These notions justify why 5-FU-DSF exerts a better antineoplastic combination than 5-FU-Cu. These parameters are indicators of programmed cell death progression and are considered the preferred way to kill cancer cells.

Nevertheless, it is also important to mention that the action of DSF/Cu essentially involves the NF-κB and TGF-β pathways during carcinogenesis and metastasis of hepatocellular carcinoma [31]. Furthermore, another study has shown that DSF-Cu combination can impede GSK3β activity via the inhibition of PARP1, resulting in immunosuppression via PD-L1 stabilization in hepatocellular carcinoma [32].

**Figure 9:**
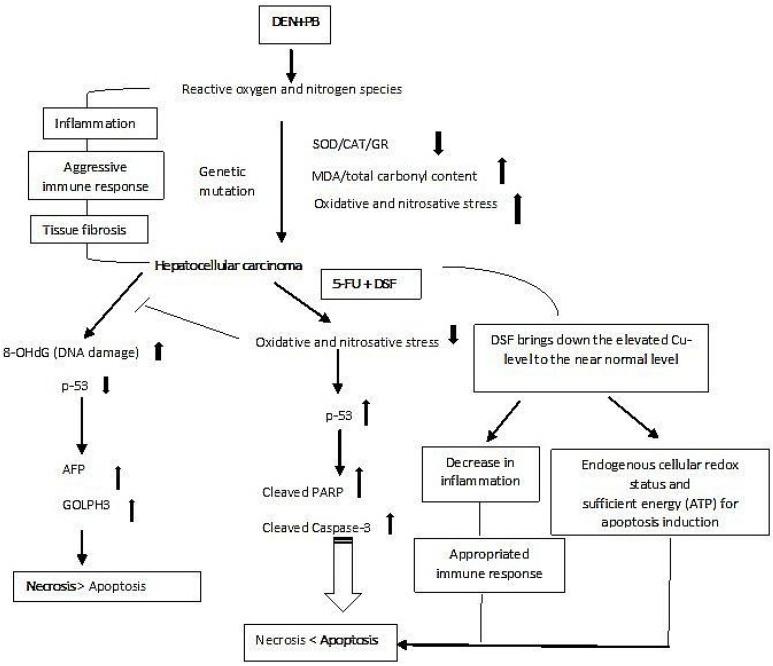
Putative mechanism of the enhanced anticancer capability of 5-FU in the presence of DSF in DEN-PB-induced hepatocarcinoma in rats. 2. Materials and Methods.

It is also possible that the marked elevation of Cu (II) in malignant cells is one of the cellular responses to handle the demand for high proliferation and angiogenesis in addition to providing a shield to nuclear chromatin to evade immune surveillance [2,33]. In the same frame of cellular events, elevated metal levels might cause the cellular machinery to scan nuclear DNA for vulnerability/damage. Depending on the integrity of the nuclear DNA, the cellular machinery decides either to halt cell cycle progression until the completion of repair of the damaged DNA or to trigger programmed cell death via the activation of caspase-7 and caspase-3 if the nuclear damage is extensive or highly uneconomic (in terms of NADH/ATP). Under such conditions, other critical proapoptotic markers, including p-53 and PARP, also facilitate the progression of apoptosis [2]. These findings also indicate that the chosen dose of the adjuvants (DSF and Cu) with 5-FU at its therapeutic dose in the combination groups engages these significant facilitators of apoptosis induction. Hitherto, both adjuvants may follow different modes of action in enhancing the antineoplastic activity of the drug. Nevertheless, DSF seems to be enjoying favoritism with the drug, as it might nullify the excessive endogenous Cu around the nuclear DNA in the chromatin, ceasing the aggression of the immune response, inflammation and oxidative stress, and allowing the cellular machinery to repair the damaged DNA or recruit facilitators of apoptosis induction and proteasome inhibition [25]. In addition, all of the excessive Cu might be chelated by DSF, which could allow 5-FU to bind to the target sites in cancer cells effectively. All these factors collectively favor DSF in improving the antineoplastic activity of 5-FU concerning Cu in the combination groups (Figure 9). Many contemporary researchers have shown similar findings that restrained management of oxidative stress by DSF/Cu has great potential to be an effective chemo- and radiotherapy treatment, with modalities addressing various forms of cancer, including cirrhosis and liver cancer [34,35].

## 4. Materials and Methods

All the materials and methodology applied have been detailed in the Supplementary File S1.

## 5. Conclusions

The present investigation indicates that the co-administration of DSF and 5-FU significantly envisages the antineoplastic activity of the drug in vivo. The chelating agent increases the sensitivity of the drug in the rats that reorient the antioxidant system and immune system (data not shown) in favor of apoptosis induction, consequently decreasing the overall tumor burden. These factors can significantly enhance cancer patients’ life span and quality of life. However, further studies are warranted to comprehend the complex array of the intricate mechanism involved in the present study.

## Data Availability

All the data relevant to the work are included in the manuscript.

## Supplementary Materials

The following supporting information can be downloaded at: https://www.mdpi.com/article/10.3390/ph16020169/s1, Supplementary File S1: Materials and Methods.
