# Supplementary material for: Disulfiram Enhances the Antineoplastic Activity and Sensitivity of Murine Hepatocellular Carcinoma to 5-FU via Redox Management
Source: Pharmaceuticals (Basel). 2023 Jan 23;16(2):169. doi: 10.3390/ph16020169 (PMC9967644; doi:10.3390/ph16020169)
Supplement: Supplementary file 1 [file pharmaceuticals-16-00169-s001.zip › pharmaceuticals-2103730-supplementary File S1.pdf]

S1. Materials and Methods

S1.1. Materials

Chemicals and Reagents

All the major chemicals of the study, including copper chloride ($CuCl_2$), 5-FU and disulfiram (DSF), were procured from Sigma-Aldrich, St. Louis, MO, USA. All the remaining reagents during different types of experimentation were purchased from Merck (Germany), BDH (England) or Sigma-Aldrich, St. Louis, MO, USA.

S1.2. Methods

1. Animal Husbandry

Seventy male and adult Swiss albino rats weighing 120 ± 20 g were obtained from the Central Animal House (Department of Pharmacy, King Saud University, Riyadh, Saudi Arabia). All rodents were reared and treated in the Departmental Animal House (Department of Zoology, King Saud University, Riyadh, Saudi Arabia) as previously [1]. All animal experiments were strictly performed according to rules framed by the Department of Zoology and the Institutional Ethical Committee of King Saud University, Riyadh.

2. Treatment with the Test Chemicals

The rodents were randomly distributed into seven treatment groups (*n* = 10), designating them from group I to VII. Group I was taken as a control negative (CN−) without any treatment. The remaining groups (II to VII) were put under cancer induction, administrating diethylnitrosamine (100 mg/kg body weight as a single intraperitoneal dose) concomitant with 0.05% (*w*/*v*) phenobarbital in their drinking water in all the groups (except the CN−) for two months. This is an established chemical(s)-induced method for hepatocellular carcinoma in rats [2,3]. After two months of cancer induction, blood samples were withdrawn from a retro-orbital region from two rats of each group and subjected to liver function tests. Their liver samples were examined under histopathology to confirm cancer induction [4, 5]. After confirmation of cancer induction, groups III, IV and V were treated with copper chloride (Cu), 5-FU and disulfiram (DSF) at standardized doses of 25, 35 and 50 mg/kg separately. Moreover, group VI was administered 5-FU and Cu, while group VII was injected with 5-FU and DSF at their respective doses. All these groups (III to VII) were given fresh water *ad libitum* during their treatment period at the frequency of twice a week for two months as per our standardized treatment strategy[2,6,7].

3. Sample Collection

In the present study, we report the death of 8 rats (5 from group II; 2 from group III and 1 from group V) among cancer-induced animals because they could not cope with the burden of the treatment despite following the safest animal handling procedures. The remaining rats at the end of the treatment were sacrificed on a single day, and their liver was washed in PBS (pH 7.36) followed by storage at −80 °C (Eppendorf, Stevenage UK). Moreover, blood samples were also collected in vacuum tubes (with EDTA),

from which serum samples were retrieved and stored at −80 °C (Eppendorf, Stevenage, UK).

The liver samples were subjected to homogenization (Ika, USA) in chilled PBS (0.1 M, pH 7.36), which was then centrifuged at 3000× *g* for 10 min (Eppendorf, Hamburg, Germany) to obtain supernatants as the tissue samples.

4. Biochemical Studies

4.1. Assessment of Cancer Markers in Serum

Alfa-feto protein (AFP) and Golgi phosphoprotein 3 (GOLPH3) are considered important biomarkers for hepatocellular carcinoma [5-8]. Their level was estimated in serum samples by commercial ELISA kits (My Biosource, Canada with catalog numbers MB S700622 and 2888376) according to the manufacturer's instructions.

4.2. Assessment of Oxidative Stress Parameters

4.2.1. Superoxide Dismutase (SOD)

Its activity was conducted by the method of Marklund and Marklund [9]. It was based on the inhibition of autooxidation of pyrogallol by the sample in succinate buffer taking absorbance every 30 s for 3 min at 412 nm on a spectrophotometer (Pharmacia Biotech, New Jersey, US).

4.2.2. Catalase Activity (CAT)

The specific activity was assessed by the protocol of H Aebi [10]. The assay was based on the degradation of hydrogen peroxide by the sample in potassium phosphate buffer every 30 s for 3 min at 412 nm on a spectrophotometer (Pharmacia Biotech, New Jersey, USA).

4.2.3. Glutathione Reductase (GR)

The assay of this enzyme was carried out by the method of Carlberg and Mannervik [11]. This method was based on oxidation of NADPH into $NADP^+$ in the presence of oxidized glutathione by the sample, and the reaction mixture was read every 30 s for 3 min at 412 nm on a spectrophotometer (Pharmacia Biotech, New Jersey, USA).

5. Estimation of Lipid Peroxidation (MDA Levels)

Measurement of lipid peroxidation by estimating malondialdehyde (MDA) levels by the standard method [12] was based on incubation of the sample with trichloroacetic acid (30%) and thiobarbituric acid (0.67%) in boiling water for 30 min. After centrifugation at 1500× *g*, the pink supernatant was read at 530 nm (Pharmacia Biotech, New Jersey, USA).

6. Measurement of Protein Oxidation (Total Carbonyl Content)

The total carbonyl content was estimated by the standard method [13] involving the usage of 2,4-dinitrophenyl hydrazine (DNPH) and guanidine hydrochloric acid. The sample was mixed with cold TCA followed by centrifugation at 4 °C for 10 min at 12,000× *g*. The pellet was further dissolved in 0.2 mL of PBS (100 mM, pH 7.4) into which 0.4 mL of DNPH (20 mM

prepared in 4 N HCl) was added. This reaction mixture was incubated for 1 h at 37 °C in a shaking water bath followed by centrifugation at 12,000× *g*; then, the pellet was further mixed with 40% TCA followed by centrifugation at 12,000× *g*. Then, the pellet formed was washed three times with a mixture of ethanol and ethyl acetate (1:1; *v*/*v*). Finally, it was dissolved in guanidine HCl (6 M), and its absorbance was observed at 360 and 280 nm against guanidine HCl as a blank (Pharmacia Biotech, New Jersey, USA).

7. Assessment of DNA Damage

8-Hydroxy-2′-deoxyguanosine (8-OHdG) is an established marker to assess the extent of DNA damage in oxidative damage-induced pathological conditions, including cancer [12,14]. According to the associated protocol, its level was estimated by a commercially available kit (BioVision, CA, USA) with an ELISA plate reader (Biochrom 2020, Cambridge, UK).

8. Assessment of Apoptotic Markers in Liver Samples

The levels of the apoptotic markers p53 and cleaved caspase-3 and cleaved PARP were estimated by commercial kits following the manufacturer’s instructions (Thermo Fisher Scientific Company, NJ, USA). All the processes from preparation of the sample to incubation with the primary and secondary antibodies followed by color development were conducted per the kits’ respective booklet. The optical densities (ODs) were measured at 405 nm by an ELISA plate reader (Biochrom, Cambridge, UK).

9. Assessment of Nuclear DNA Damage in Liver Cells

Comet assay is a very reliable experiment to assess damage to nuclear DNA in vivo or in situ. It was conducted as per our lab’s standardized procedure [2,6].

10. Assessment of Necrosis

Lactate dehydrogenase (LDH) is an indirect parameter for assessment of necrosis in the treated cells [2]. The measurement of its activity was conducted in the tissue samples by the commercial kit (QCA, Tarragona, Spain).

11. Histopathology of the Liver Samples

The samples from all the treatment groups were preserved in 8% formalin to fix them for histopathology studies. Their paraffin embedding was conducted in 10 × 5 × 3 mm tissue blocks followed by section cutting of 5–7 μm thickness by a rotary microtome. The prepared sections were stained with hematoxylin and eosin dye. The sections were studied using a standard microscope (DM IL LED, Leica, Munich, Germany), and pictures were taken at a magnification of 400× by the attached camera (Leica, Munich, Germany).

S1.3. *Statistical Analysis*

All the experimental data are expressed as the mean ± SEM analyzed by GraphPad Prism 5 software. The data analysis was carried out by one-way ANOVA with Tukey’s post hoc, assuming p-value < 0.05 as statistically significant with the help of the software. The asterisk marks * and # indicate

significant differences from the negative control (group I) and positive control (group II) in vivo studies as calculated by Student's t-test. The asterisks ** and ## indicate $p < 0.01$, while *** and ### indicate $p < 0.001$.

References:

1. Alhazza, I.M.; Ebaid, H.; Omar, M.S.; Hassan, I.; Habila, M.A.; Al-Tamimi, J.; Sheikh, M. Supplementation with selenium nanoparticles alleviates diabetic nephropathy during pregnancy in the diabetic female rats. *Environ. Sci. Pollut. Res.* **2022**, *29*, 5517–5525.
2. Hassan, I.; Khan, A.A.; Aman, S.; Qamar, W.; Ebaid, H.; Al-Tamimi, J.; Alhazza, I.M.; Rady, A.M. Restrained management of copper level enhances the antineoplastic activity of imatinib in vitro and in vivo. *Sci. Rep.* **2018**, *8*, 1–17.
3. Tabrez, S.; Priyadarshini, M.; Urooj, M.; Shakil, S.; Ashraf, G.M.; Khan, M.S.; Kamal, M.A.; Alam, Q.; Jabir, N.R.; Abuzenadah, A.M. Cancer chemoprevention by polyphenols and their potential application as nanomedicine. *J. Environ. Sci. Health Part C* **2013**, *31*, 67–98.
4. Ding, Y.-f.; Wu, Z.-h.; Wei, Y.-j.; Shu, L.; Peng, Y.-r. Hepatic inflammation-fibrosis-cancer axis in the rat hepatocellular carcinoma induced by diethylnitrosamine. *J. Cancer Res. Clin. Oncol.* **2017**, *143*, 821–834.
5. Sun, H.; Yu, L.; Wei, H.; Liu, G. A novel antihepatitis drug, bicyclol, prevents liver carcinogenesis in diethylnitrosamine-initiated and phenobarbital-promoted mice tumor model. *J. Biomed. Biotechnol.* **2012**, *2012*, 584728.
6. Hassan, I.; Chibber, S.; Khan, A.A.; Naseem, I. Riboflavin ameliorates cisplatin induced toxicities under photoillumination. *PLoS One* **2012**, *7*, e36273.
7. Khan, A.A.; Jabeen, M.; Khan, A.A.; Owais, M. Anticancer efficacy of a novel propofol–linoleic acid-loaded escheriosomal formulation against murine hepatocellular carcinoma. *Nanomedicine* **2013**, *8*, 1281–1294.
8. Liu, H.; Wang, X.; Feng, B.; Tang, L.; Li, W.; Zheng, X.; Liu, Y.; Peng, Y.; Zheng, G.; He, Q. Golgi phosphoprotein 3 (GOLPH3) promotes hepatocellular carcinoma progression by activating mTOR signaling pathway. *BMC Cancer* **2018**, *18*, 1–12.
9. Marklund, S.; Marklund, G. Involvement of the superoxide anion radical in the autoxidation of pyrogallol and a convenient assay for superoxide dismutase. *Eur. J. Biochem.* **1974**, *47*, 469–474.
10. Aebi, H. [13] Catalase in vitro. In *Methods in Enzymology*; Elsevier: Amsterdam, The Netherlands, 1984; Volume 105, pp. 121–126.
11. Carlberg, I.; Mannervik, B. [59] Glutathione reductase. In *Methods in Enzymology*; Elsevier: Amsterdam, The Netherlands, 1985; Volume 113, pp. 484–490.
12. Buege, J.; Aust, S. Microsomal lipid peroxidation. *Methods Enzymol*. **1978**, *52*, 302–310.
13. Levine, R.L.; Williams, J.A.; Stadtman, E.P.; Shacter, E. [37] Carbonyl assays for determination of oxidatively modified proteins. In *Methods in Enzymology*; Elsevier: Amsterdam, The Netherlands, 1994; Volume 233, pp. 346–357.
14. Valavanidis, A.; Vlachogianni, T.; Fiotakis, C. 8-hydroxy-2′-deoxyguanosine (8-OHdG): A critical biomarker of oxidative stress and carcinogenesis. *J. Environ. Sci. Health Part C* **2009**, *27*, 120–139.
